# rs9789446 genotype as susceptibility biomarkers for congenital hypothyroidism based on population and family validation

**DOI:** 10.3389/fgene.2025.1721264

**Published:** 2025-12-18

**Authors:** Xue Zhong, Yaning Jia, Wenmiao Liu, Mengfei Qiao, Miaomiao Li, Xiaolong Yu, Shiguo Liu

**Affiliations:** 1 Medical Genetic Department, The Affiliated Hospital of Qingdao University, Qingdao, China; 2 Prenatal Diagnosis Center, The Affiliated Hospital of Qingdao University, Qingdao, China; 3 Medical Record Department, Weifang People’s Hospital, Weifang, China; 4 Department of Endocrinology, The Affiliated Hospital of Qingdao University, Qingdao, China

**Keywords:** congenital hypothyroidism, endocrinology, genetics, genotype, polymorphism

## Abstract

**Introduction:**

Congenital hypothyroidism (CH) is a metabolic disorder in newborns due to insufficient synthesis, abnormal secretion, or defective action of thyroid hormones. While newborn screening enables early detection, the precise etiology remains elusive in most cases, with genetic factors playing a crucial but incompletely characterized role. This study comprehensively investigated the association of the rs9789446 polymorphism with CH risk, its interactions with biological sex and clinical subtypes, and its impact on thyroid function severity.

**Methods:**

A case-control study was conducted with 306 CH patients and 441 controls. Genotyping for rs9789446 was performed using SNPscan™. Association analyses included chi-square tests, logistic regression stratified by biological sex and clinical features, linear regression for thyroid parameters, and family-based validation in 201 trios using TaqMan™ assays.

**Results:**

The minor G allele frequency was significantly lower in CH patients (0.348) than in controls (0.407). A protective association was observed for the G allele against CH risk (OR = 0.78, p = 0.021), with a stronger effect in males under the dominant model (OR = 0.57, p = 0.008) but no association in females, highlighting a pronounced sex-specific effect. Stratification by permanent or temporary subtypes showed no significant association, while a modest effect was detected in the goitrous subgroup under the dominant model. Initial thyroid hormone levels exhibited no significant correlation. Importantly, family-based analyses robustly validated the case-control findings.

**Discussion:**

The rs9789446 G allele confers a sex-specific protective effect against CH, particularly in males. This supports its potential utility in genetic risk assessment and personalized screening strategies for early intervention.

## 1 Introduction

Congenital hypothyroidism (CH) is the most common endocrine disorder in newborns (Park, 2021; Arrigoni et al., 2025), with primary CH occurring at an incidence of approximately 1 in 3000 to 1 in 2000 live births (Van Trotsenburg et al., 2021). A notable biological sex disparity exists, with female patients outnumbering males by a ratio of roughly 2:1 (Uthayaseelan et al., 2022). Despite newborn screening (NBS) programs having facilitated early detection and intervention for over 40 years, significant challenges remain in our understanding of thyroid morphology and function for complex etiologies (Cavarzere et al., 2025). The condition exhibits substantial clinical heterogeneity, including both permanent and temporary forms (Dermitzaki et al., 2025), as well as thyroid morphological manifestations that vary widely, ranging from athyreosis and thyroid ectopy to dyshormonogenesis (Abduljabbar and Afifi, 2012).

The genetic underpinnings of CH encompass thyroid dysgenesis (TD) and thyroid dyshormonogenesis (TDH) (Tsai et al., 2024). Over the year, several candidate genes have been implicated: *PAX8*, *NKX2-1*, *FOXE1*, *NKX2-5,* and *HHEX* for TD pathogenesis, and *DUOX2*, *DUOXA2*, *DUOX1*, *TPO*, *TG*, *SLC26A4*, *SLC5A5*, and *TSHR* for TDH (Sun et al., 2018; Ahn and Jeong, 2025; Du et al., 2025; Uehara et al., 2024). However, the precise pathogenesis of CH remains complex and incompletely elucidated. A recent genome-wide association study (GWAS) identified an association between the gene locus 2q33.3 (rs9789446) and TD in various ethnic populations (Narumi et al., 2022).

To investigate the rs9789446 polymorphism’s association with CH susceptibility, we employed a multifaceted genetic association study design incorporating (Park, 2021): case-control association analysis to evaluate allele frequency differences (Arrigoni et al., 2025); clinical stratification by disease etiology (TD vs. TDH) and thyroid morphological characteristics (Van Trotsenburg et al., 2021); genotype-phenotype relationship analyses utilizing biochemical severity indicators; and (Uthayaseelan et al., 2022) family-based validation through transmission disequilibrium testing. This comprehensive analytical framework provides novel insights into the genetic architecture of CH, particularly regarding how common genetic variants may influence thyroid gland development and hormonal biosynthesis pathways, potentially improving future risk stratification protocols and personalized therapeutic approaches for CH management.

## 2 Materials and methods

### 2.1 Study subjects

All study subjects were Chinese Han individuals recruited from major hospitals and newborn screening centers in multiple cities of China (including Qingdao, Jinan, Tai’an, Liaocheng and Xuzhou) during the period from 2014 to 2024. For this case-control study, 306 CH patients and 441 healthy controls were recruited. Newborns with thyroid-stimulating hormone (TSH) screening results >9 mIU/L measured within 48–72 h after birth, underwent repeat testing at 1–3 weeks to verify serum levels with high TSH (>7.63 mIU/L) and low free tetraiodothyronine (FT4) (<11.5 pmol/L) were verified (Jia et al., 2025). The exclusion criteria comprised other endocrine, genetic metabolic diseases, and congenital disorders, such as chromosomal abnormalities or related syndromes, as well as positive serum anti-thyrotropin receptor antibodies. Thyroid morphology was assessed via ultrasound or technetium-99 m scanning. The control subjects, matched for age and ethnic background, were healthy individuals with no personal or family history of thyroid disorders. Based on this, we successfully collected 201 complete trios consisting of father, mother and child (i.e., the patient and their biological parents) from these 306 cases, for conducting family-based tests.

### 2.2 Clinical data collection

Detailed clinical data were collected for all patients, including age at enrollment, biological sex, TSH, FT4, initial and maximum adjusted levothyroxine (L-T4) replacement doses, and etiological classification (permanent or temporary CH, goitrous or non-goitrous). Goiter was determined via thyroid ultrasound.

### 2.3 Genotyping

Venous blood samples (3–4 mL) were collected from the median cubital vein of the study subjects and stored in the vacuum vasculature of EDTA anticoagulation. The genomic DNA was isolated using the TIANamp Blood DNA Kit (TIANGEN BIOTECH, Beijing, China) according to the directions from the peripheral blood samples.

The samples for the case-control study were genotyped for rs9789446 using a custom-designed SNPscanTM kit (provided by Shanghai Genesight Biotechnology Co., Ltd.) through a dual-ligation reaction and multiplex fluorescence polymerase chain reaction method. Subsequently, these genotypes were further verified by Sanger sequencing. We excluded the samples with a genotype accuracy rate lower than 95%. Overall, the accuracy rate of genotype determination was 97.94%.

**FIGURE 1:**
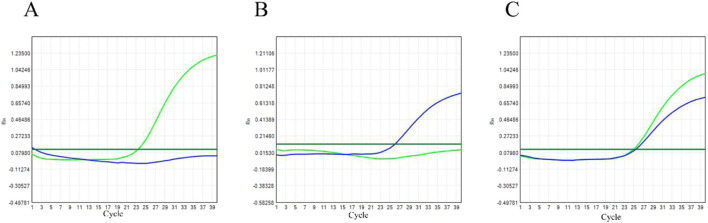
Representative real-time PCR amplification curves for genotyping the rs9789446 polymorphism using the TaqMan SNP Genotyping Assay. **(A)** Genotype of AA type. **(B)** Genotype of GG type. **(C)** Genotype of AG type.

The genotyping of the family study samples was performed using TaqMan allelic discrimination real-time PCR (Figure 1). The manufacturer of the TaqMan^TM^ SNP Genotyping Assays Kit is Appliedbiosystems by Thermo Fisher Scientific. After SNP system verification, the test dose for 211 nuclear family trios with CH was as follows: TaqMan probe 0.2 µL, PCR Master MIX 12.5 µL, double-distilled water 9.8 µL, and DNA 2.5 µL. Procedure: program segment 1 (95 °C for 10 min, cycle number 1), program segment 2 (95 °C for 15 s, 60 ° C for 1 min, cycle number 40). The genotype of each sample could be identified by detecting the fluorescent signal from VIC- or FAM-labeled probes in each cycle. The Mendelian error check performed on all 201 trios, confirming full consistency. In addition, 12 subjects with inconclusive TaqMan assays' real-time results were selected for DNA sequencing techniques to determine genotype. The forward primer sequence is 5′-TTC​CCT​AGC​CTC​CTG​TCC​ACT​A-3′. The reverse primer sequence is 5′-CCT​GGC​CAT​CTG​GGT​CAG​T-3′.

### 2.4 Statistical analysis

All statistical analyses were performed using PLINK software (v1.9) (http://zzz.bwh.harvard.edu/plink/) and SPSS Statistics (v27.0). The control group was assessed for Hardy-Weinberg equilibrium (HWE) using a Chi-square test. For the case-control study, the difference in allele frequencies between cases and controls was evaluated using the chi-square test. Logistic regression analysis was conducted using additive, dominant and recessive genetic models to calculate the odds ratio (OR) and 95% confidence interval (CI). These analyses were adjusted for age and biological sex as specified covariates. The stratified analysis was repeated in the subgroups stratified by biological sex, etiology (permanent or temporary hypothyroidism), and thyroid morphology (large goiter or non-goiter large thyroid). In the quantitative trait analysis within the patient population, the association of the rs9789446 genotype and continuous variables (initial thyroid-stimulating hormone level, free thyroxine level) was examined under different models using a linear regression model. For family-based analysis, we assessed three complementary methods in 201 nuclear family trios using the transmission disequilibrium test (TDT), the haplotype-based haplotype relative risk (HHRR) test, and the haplotype relative risk (HRR) test. Statistical significance was considered to be present when the p-value was less than 0.05. Given the multiple statistical tests performed across different genetic models and stratified analyses, we implemented a tiered False Discovery Rate (FDR) correction strategy using the Benjamini–Hochberg procedure to control for false positives. A conventional FDR significance threshold of q < 0.05 was applied to declare statistical significance.

## 3 Results

### 3.1 Basic information and genetic distribution

The chromosomal location of rs9789446 is at 208899346 on chromosome 2 with the common allele A and the minor allele G. The genotype distribution of rs9789446 in the control group was in accordance with the Hardy-Weinberg equilibrium (HWE) (*p* = 0.8165), indicating that this was a representative sample group. The frequency of the minor allele (G) in the cases (0.348) was significantly lower than that in the controls (0.407), suggesting that this allele may have a potential protective effect. Table 1 provides the clinical characteristics of 747 participants in the case-control study. This study included 306 patients with CH (148 males and 158 females) and 441 healthy controls (257 males and 184 females). The age of the case group was 8.85 ± 3.71 years, and that of the control group was 8.44 ± 3.14 years. The two groups were well-matched in age (*p* = 0.072). The subsequent logistic regression model adjusted for biological sex and age as covariates.

**TABLE 1:** General characteristics of congenital hypothyroidism cases and healthy subjects.

Variables	Cases (n = 306)		Controls (n = 441)		*p*-value
Age, years (mean ± SD)	8.850 ± 3.711		8.444 ± 3.143		0.072
TSH (mIU/L) (mean ± SD)	61.157 ± 70.020		2.412 ± 1.063		**0.001**
FT4 (pmol/L) (mean ± SD)	5.975 ± 5.780		14.561 ± 3.606		**0.001**
Initial L-T4 dose (μg/day) (mean ± SD)	27.220 ± 5.600		​	​	​
Maximum adjusted dose (μg/day) (mean ± SD)	42.247 ± 17.535		​	​	​
​	Male (n = 148)	Female (n = 158)	Male (n = 257)	Female (n = 184)	​
Age, years (mean ± SD)	8.480 ± 3.061	8.411 ± 3.228	8.755 ± 3.589	8.984 ± 3.880	​
TSH (mIU/L) (mean ± SD)	48.642 ± 56.789	72.880 ± 78.861	2.417 ± 1.073	2.405 ± 1.052	​
FT4 (pmol/L) (mean ± SD)	5.063 ± 3.509	7.407 ± 20.615	14.679 ± 3.545	14.396 ± 3.694	​
Initial L-T4 dose (μg/day) (mean ± SD)	26.692 ± 5.328	27.715 ± 5.816	​	​	​
Maximum adjusted dose (μg/day) (mean ± SD)	41.836 ± 19.108	42.632 ± 15.973	​	​	​

Data are presented as mean ± standard deviation (SD). Group comparisons for continuous variables between cases and controls were performed using the independent samples t-test for normally distributed data (Age) and the Mann-Whitney U test for non-normally distributed data (TSH, FT4). L-T4, levothyroxine. Bold text in the *p*-value column indicate statistically significant differences (*p* < 0.05).

### 3.2 Overall genetic association analysis

A comprehensive case-control association analysis was conducted. The allele-based analysis revealed a protective trend for the minor G allele against CH (OR = 0.778, 95% CI 0.628–0.963, *p* = 0.021), though this did not survive FDR correction (q = 0.084). Subsequent logistic regression analysis under dominant, recessive, and additive genetic models—adjusted for biological sex and age—further clarified this association. Collectively, the results indicated a significant association between the rs9789446 G allele and a reduced risk of CH. Under the dominant genetic model (GG + AG vs. AA), carriers of at least 1 G allele showed significantly reduced CH risk compared to AA homozygotes (OR = 0.710, 95% CI 0.525–0.960, *p* = 0.026, FDR q = 0.035). The recessive model (GG vs. AA + AG) revealed no statistically significant association (OR = 0.752, 95% CI 0.493–1.148, *p* = 0.187, FDR q > 0.05), suggesting that the protective effect does not require homozygosity for the G allele. Furthermore, the additive model analysis, which examines the linear effect of each additional G allele, confirmed a significant dose-dependent protective relationship (OR = 0.781, 95% CI 0.630–0.969, *p* = 0.025, FDR q = 0.050).

These findings are summarized in Table 2.

**TABLE 2:** Analysis of genotypes of rs9789446.

Model	Cases (n)	Controls (n)	OR (95% CI)	*p*-value	q-value
Allele					
A	399	523	1 (reference)	​	​
G	213	359	0.778 (0.628, 0.963)	**0.021**	**0.084**
Dominant					
AA	132	154	1 (reference)	​	​
GG + AG	174	287	0.710 (0.525, 0.960)	**0.026**	**0.035**
Recessive					
GG	39	72	1 (reference)	​	​
AA + AG	267	369	0.752 (0.493, 1.148)	0.187	0.187
Additive	-	-	0.781 (0.630, 0.969)	**0.025**	**0.050**

OR: odds ratio, representing the odds of disease in one group compared to the reference group. An OR <1 indicates a protective effect of the minor G allele against congenital hypothyroidism. 95% CI: 95% Confidence Interval, indicating the precision of the OR, estimate. Genetic models: The models are defined with respect to the minor G allele, which demonstrated a protective effect in our study. The ‘n' for the Allele model denotes allele counts, while for the Genotype models (Dominant, Recessive) it denotes the number of individuals. (Park, 2021) Allele: Compares the frequency of G vs. A alleles. (Arrigoni et al., 2025) Dominant: Combines GG, and AG, genotypes versus AA genotype. (van Trotsenburg et al., 2021) Recessive: Compares GG, genotype versus AA and AG, genotypes combined. (Uthayaseelan et al., 2022) Additive: Assumes a linear effect of each additional G allele. Association analyses were performed using chi-square test for allele frequencies and logistic regression for genetic models (additive, dominant, recessive), adjusting for age and sex where appropriate. P-value was corrected by False Discovery Rate (FDR) correction. Statistical significance was defined as FDR q-value <0.05, with significant values shown in bold.

### 3.3 Sex-stratified association analysis

Given the observed biological sex distribution differences and the clear biological sex-specific characteristics of CH, we stratified the study population by biological sex to explore possible biological sex-specific genetic effects. This analysis revealed significant and stable biological sex differences. In the male subgroup, this association was highly significant, and the dominant model revealed the strongest effect, indicating that male carrying the G allele had a significantly lower risk of CH (OR = 0.566, 95% CI 0.371–0.862, *p* = 0.008, FDR q = 0.032). The additive model also demonstrated a protective trend (OR = 0.733, 95% CI 0.541–0.994, *p* = 0.045, FDR q = 0.091). In contrast, in the female subgroup, no significant association was detected under any genetic model (all *p* > 0.05). The complete results of the biological sex stratification analysis are detailed in Table 3, emphasizing that the protective effect of the rs9789446 G allele is limited to male.

**TABLE 3:** Analysis of genotypes of rs9789446 among male and female.

Model	Male					Female				​
	Cases (n)	Controls (n)	Or (95% CI)	*p*-value	q-value	Cases (n)	Controls (n)	OR (95% CI)	*p*-value	q-value
Allele										
A	192	298	1 (reference)	​	​	207	225	1 (reference)	​	​
G	104	214	0.747 (0.556, 1.005)	0.053	0.071	109	143	0.829 (0.606,1.133)	0.238	0.476
Dominant										
AA	65	80	1 (reference)	​	​	67	74	1 (reference)	​	​
GG + AG	83	176	0.566 (0.371, 0.862)	**0.008**	**0.032**	91	110	0.906 (0.587,1.397)	0.655	​
Recessive										
GG	21	38	1 (reference)	​	​	18	33	1 (reference)	​	​
AA + AG	127	218	0.928 (0.523, 1.648)	0.799	0.799	140	151	0.597 (0.321,1.110)	0.103	0.412
Additive	-	-	0.733 (0.541, 0.994)	**0.045**	0.091	-	-	0.836 (0.616,1.14)	0.252	0.336

Sex-stratified association analyses were performed separately for males and females. Allele frequencies were compared using chi-square tests. Genetic models (additive, dominant, recessive) were analyzed using logistic regression adjusted for age. P-value was corrected by False Discovery Rate (FDR) correction. Statistical significance was defined as FDR q-value <0.05, with significant values shown in bold.

### 3.4 Analysis stratified by clinical subtypes

To investigate potential differences in genetic associations across distinct manifestations of CH, we performed comprehensive analyses stratified by disease etiology and thyroid morphology. First, we categorized cases into permanent CH (PCH) (n = 218) and we categorized cases into temporary CH (TCH) (n = 88) subgroups. Genetic analysis revealed no significant association between the rs9789446 polymorphism and either CH subtype across all genetic models when compared with controls (all *p* > 0.05, Table 4). Subsequently, patients were stratified based on goiter status into goitrous (n = 183) and non-goitrous (n = 123) groups. Analysis within the goitrous cohort revealed protective trends that approached, but did not reach, formal statistical significance after multiple testing correction (dominant model: OR = 0.689, *p* = 0.040; additive model: OR = 0.774, *p* = 0.049; both FDR q > 0.05). No significant associations were detected in the non-goitrous group (Table 4). These findings suggest potential differential genetic contributions to CH pathogenesis based on clinical presentation, particularly regarding goiter formation.

**TABLE 4:** Analysis of the rs9789446 genotype for permanent congenital hypothyroidism (PCH) or temporary congenital hypothyroidism (TCH), as well as for goiter or non-goiter.

Model	Cases (n)	Controls (n)	Or (95% CI)	*p*-value (q-value)	Cases (n)	Controls (n)	Or (95% CI)	*p*-value (q-value)
​	PCH				TCH			
Allele								
A	280	523	1 (reference)	​	117	523	1 (reference)	​
G	156	359	0.812 (0.640, 1.029)	0.085 (0.17)	59	359	0.735 (0.523, 1.033)	0.075 (0.300)
Dominant								
AA	93	154	1 (reference)	​	39	154	1 (reference)	​
GG + AG	125	287	0.714 (0.509, 1.001)	0.051 (0.204)	49	287	0.692 (0.434, 1.104)	0.123 (0.164)
Recessive								
GG	31	72	1 (reference)	​	10	72	1 (reference)	​
AA + AG	187	369	0.856 (0.539, 1.359)	0.510 (0.510)	78	369	0.646 (0.319, 1.310)	0.226 (0.226)
Additive	-	-	0.812 (0.639,1.032)	0.089 (0.119)	-	-	0.743 (0.527, 1.045)	0.088 (0.176)
​	Goiter				Non-goiter			
Allele								
A	238	523	1 (reference)	​	161	523	1 (reference)	​
G	128	359	0.784 (0.608, 1.009)	0.059 (0.079)	85	359	0.769 (0.573, 1.033)	0.081 (0.324)
Dominant								
AA	79	154	1 (reference)	​	53	154	1 (reference)	​
GG + AG	104	287	0.689 (0.483, 0.983)	**0.040** (0.159)	70	287	0.751 (0.497, 1.135)	0.174 (0.232)
Recessive								
GG	24	72	1 (reference)	​	15	72	1 (reference)	​
AA + AG	159	369	0.771 (0.467,1.272)	0.309 (0.309)	108	369	0.718 (0.394, 1.310)	0.280 (0.280)
Additive	-	-	0.774 (0.599, 1.000)	**0.049** (0.098)	-	-	0.795 (0.590,1.071)	0.131 (0.262)

Sex-stratified association analyses were performed separately for males and females. Allele frequencies were compared using chi-square tests. P-value was corrected by False Discovery Rate (FDR) correction. Statistical significance was defined as FDR q-value <0.05, with significant values shown in bold.

### 3.5 Association with biochemical severity

We investigated the relationship between the rs9789446 genotype and the biochemical severity of CH at diagnosis within the patient cohort. Linear regression analyses, under additive, dominant, and recessive models, were employed to assess their effect on initial TSH and FT4 levels. No significant associations were observed between the different genotypes and any of the thyroid function parameters (all *p* > 0.05, Table 5), indicating that while this locus influences susceptibility to CH, it does not appear to modulate the biochemical severity of the disease at presentation.

**TABLE 5:** Association of rs9789446 with thyroid function parameters in congenital hypothyroidism cases.

Variable	Model	β (95% CI)	*p*-value	q-value	Adjusted R^2^
TSH	Dominant	2.950 (-12.976, 18.877)	0.716	0.716	−0.003
	Recessive	20.127 (-3.422, 43.677)	0.094	0.282	0.006
	Additive	6.350 (-5.179, 17.880)	0.279	0.419	0.001
FT4	Dominant	−0.787 (-2.099, 0.525)	0.239	0.717	0.001
	Recessive	−0.541 (-2.493, 1.411)	0.586	0.586	−0.002
	Additive	−0.543 (-1.495, 0.409)	0.262	0.393	0.001

Association analyses were performed using linear regression within the congenital hypothyroidism case cohort (n = 306) to assess the relationship between rs9789446 genotypes and thyroid function parameters at diagnosis. A total of three genetic models (dominant, recessive, and additive) were tested, and all of them were adjusted for age and biological sex. P-value was corrected by False Discovery Rate (FDR) correction. Statistical significance was defined as FDR q-value <0.05. Adjusted R^2^ indicates the proportion of variance in the thyroid function parameter explained by the genetic model after adjusting for age and sex.

### 3.6 Family-based association analysis

To validate our case-control findings and provide independent evidence for the association, we conducted a family-based study using 201 case-parent trios. Among them, 108 were heterozygous mothers and 111 were heterozygous fathers. The family-based approach controls for population stratification bias. The TDT yielded a statistically significant result (OR = 0.607, 95% CI 0.396–0.929, *p* = 0.031, FDR q = 0.047), which evaluated the preferential transmission of alleles from heterozygous parents to affected offspring. However, there was no significant association observed between rs9789446 and CH for HRR (OR = 1.522, 95% CI 0.831–2.786, *p* = 0.171, FDR q > 0.05). To improve the efficiency of the test, we performed the HHRR analysis, which effectively expanded the statistical power of cases. We observed a trend of association for this polymorphism (OR = 1.402, 95% CI 1.058–1.858, *p* = 0.018, FDR q = 0.054). The concordance between the case-control and family-based results (TDT and HHRR) provides robust and complementary evidence for the genuine association between the rs9789446 G allele and a reduced risk of developing CH. These results are presented in Table 6.

**TABLE 6:** The results of a family-based association in 211 trios.

Model					χ2	*p*-value	q-value	Or (95% CI)
TDT	​	​	Non-transmitted allele		​	​	​	​
	​	​	A	G	​	​	​	​
	Transmitted allele	A	158	126	​	​	​	​
		G	93	45	4.676	**0.031**	**0.047**	0.607 (0.396–0.929)
HRR	​	​	A+	A-	​	​	​	​
	Transmitted allele	​	191	20	​	​	​	​
	Non-transmitted allele	​	182	29	1.87	0.171	0.171	1.522 (0.831–2.786)
HHRR	​	Transmitted allele						
	Non-transmitted allele	​	A	G	​	​	​	​
	​	​	284	138	5.56	**0.018**	0.054	1.402 (1.058–1.858)

The Chi-square test was used to conduct TDT, HRR, and HHRR, analyses on the family. P-value was corrected by False Discovery Rate (FDR) correction. Statistical significance was defined as FDR q-value <0.05, with significant values shown in bold.

## 4 Discussion

CH is the most frequent endocrine disorder in newborns and a leading cause of preventable intellectual disability (Danner et al., 2023). Newborns with CH are often asymptomatic or present mild non-specific symptoms such as prolonged jaundice, feeding difficulties, hoarse crying, constipation, umbilical hernia, and macroglossia (Rastogi and LaFranchi, 2010). Without timely and adequate L-T4 treatment during childhood, they may develop irreversible intellectual disability and dwarfism (Long et al., 2021). Although NBS programs have been highly successful in enabling early treatment and preventing severe cognitive deficits, the underlying pathogenic mechanisms remain largely unknown in a substantial number of cases (Klosinska et al., 2022). It is widely accepted that genetic factors contribute critically to the pathogenesis of CH (Zhang et al., 2025). To date, more than 29 causative genes have been implicated in its etiology (Wang et al., 2020). However, these mutations only explain only a small fraction of cases, indicating that common genetic variations and complex genetic patterns play a significant role in this process. GWAS and candidate gene approaches have identified several susceptibility loci, offering new insights into the polygenic nature of the disease (Zucker et al., 2024). Narumi et al. (2022) performed the first GWAS and identified rs9789446 as a risk for TD in Japanese populations, and replicated this association in German populations using a sporadic case-control association study (Narumi et al., 2022). However, the specific role of rs9789446 in CH in the Chinese Han population has not been thoroughly investigated. A comprehensive analysis of this variant, particularly regarding its potential regulatory effect on clinical manifestations and severity, is currently lacking in relevant research. Furthermore, a compelling aspect of CH epidemiology is its well-documented female predominance, hinting at potential sex-linked or sex-modified genetic factors that have been largely unexplored in genetic association studies.

This comprehensive investigation identified and validated a significant association between the rs9789446 polymorphism and a reduced risk of CH, revealing a complex pattern of genetic susceptibility that advances our understanding of CH pathogenesis. Our multi-tiered analytical framework, which incorporated both case-control and family-based designs, provides robust evidence for a protective role of the minor G allele, while simultaneously uncovering intriguing sex-specific effects that merit careful interpretation. The core finding of a protective association gains particular biological plausibility when considered in the context of the genomic region surrounding rs9789446. The reduced frequency of the protective G allele in patients suggests it may confer enhanced regulatory efficiency in thyroid morphogenesis or hormone biosynthesis pathways. This finding aligns with emerging recognition that common genetic variants clarify a key genetic determinant in CH (Jia et al., 2025).

Our sex-stratified analysis revealed a pronounced male-specific protective effect that was completely absent in females. This observed sexual dimorphism may be explained by developmental programming mechanisms. Studies in rodent models indicate that the thyroid function status during the embryonic and neonatal periods determines the expression level and signaling capacity of androgen receptors in the adult prostate (Aruldhas et al., 2010). This developmental programming effect provides a plausible explanation for our findings: the protective rs9789446 G allele may maintain more ideal thyroid hormone levels during the critical developmental window, which in turn could promote a more robust establishment of the androgen signaling pathway specifically in males. Subsequently, enhanced androgen receptor expression or function may confer stronger recovery ability to cope with developmental disorders that cause CH, thereby manifesting as the male-specific protective effect. The developmental interaction between the thyroid and androgen may explain why the significant association was confined to males, as the developing prostate and male reproductive system have a special dependence on androgen signals during normal development (Bhagwat and Vakoc, 2015).

The lack of association when stratified by permanent versus temporary etiology suggests that rs9789446 influences a common pathogenic mechanism relevant to both forms of CH. This concept finds support in the established genetics of CH, where variations in genes like *DUOX2* and *DUOXA2* can present as either PCH or TCH hypothyroidism (Peters et al., 2019). Akin to these genes, rs9789446 may affect a fundamental process whose perturbation can manifest across the clinical spectrum.

Furthermore, the weak association observed specifically in the goitrous subgroup under the dominant model hints that this variant might have particular relevance for thyroid growth regulation pathways. While goitrogenesis is commonly attributed to TSH-driven hyperplasia (Djerbib et al., 2025), other pathways involving growth factors (e.g., IGF-1) (Smith and Pasternak, 2025) and specific genetic defects (e.g., in TG) (Cevizoglu et al., 2025) are also well-established. The rs9789446 variant may therefore not directly affect primary hormone synthesis but could instead modulate secondary compensatory responses, potentially influencing the threshold for or the magnitude of glandular hyperplasia in the face of hormonal deficiency.

In contrast, our negative findings regarding biochemical severity parameters (TSH, FT4) suggest that while rs9789446 influences disease susceptibility, it does not substantially modulate disease severity among affected individuals. This distinction between susceptibility and severity loci is well-recognized in complex disorders (El-Toukhy, 2015) and implies that different genetic factors may govern these two aspects of CH pathogenesis.

Population-based case-control studies offer practical advantages, such as straightforward sample identification and lower costs, assuming a relatively even population distribution. However, they are susceptible to confounding from population stratification, which can inflate test statistics and lead to false-positive associations when genetic heterogeneity is present (Hirschhorn et al., 2002; Ioannidis et al., 2011). To reduce the effect of population stratification, we complemented the case-control analysis with a family-based association study including TDT, HRR, and HHRR analyses. The TDT method, which uses parental genotypes as internal controls, is largely robust to population stratification and can yield more reliable results (Liu W. et al., 2022). However, due to its low statistical power especially for low allele frequencies, it is difficult to achieve statistical significance without using a large number of families, which is prone to false negatives (Liu K. et al., 2022). Given that both study designs have complementary strengths and limitations, we integrated them to minimize stratification bias while enhancing overall statistical robustness. Therefore, to validate the results of the case-control study, we conducted a TDT based on 201 trios. The significant TDT results indicating that protective alleles are more likely to be passed from heterozygous parents to unaffected children, provide evidence at the family level for the initial association. Additionally, our case-control study lacks whole-genome data, which prevents us from conducting formal genetic correlation checks and performing complex ancestral control through principal component analysis. However, we have further corroborated the key findings using a family-based analysis (TDT) within a subset of the cases. This method, which is inherently robust to population stratification and other confounding factors, provides orthogonal validation and strengthens the evidence for a true genetic association. Several limitations warrant careful consideration. First, although our sample size was substantial, it had limited statistical power for detecting modest effects in subgroup analyses. Second, the precise functional mechanism through which rs9789446 exerts its effect remains unknown—it may be in linkage disequilibrium with a causal variant rather than being functional itself. Third, potential gene-gene and gene-environment interactions were beyond the scope of this study but represent important directions for future research. Despite these limitations, our findings have meaningful implications. The identification of a sex-specific genetic effect underscores the importance of considering sex as a biological variable in genetic studies of CH. From a clinical perspective, if validated in independent cohorts, genetic markers like rs9789446 could eventually contribute to improved risk prediction models, particularly for male infants.

## 5 Conclusion

Through case-control studies and family-based analyses, this research has confirmed that the rs9789446 polymorphism is significantly associated with CH in the Chinese Han population, and it has also for the first time demonstrated a male-specific protective effect. These findings highlight the potential of including rs9789446 genotyping in genetic risk assessment models, which can help stratify risks, especially for male infants, and provide information for more personalized and potentially earlier screening strategies to improve intervention effectiveness. Future research should focus on elucidating the underlying biological mechanisms behind this biological sex-specific effect and exploring the interactions between this locus and other genetic and environmental factors in larger and more diverse populations.

## Data Availability

The raw data supporting the conclusions of this article will be made available by the authors, without undue reservation.
